# Malignant Cells Beyond the Tumor Core: The Non‐Negligible Factor to Overcome the Refractory of Glioblastoma

**DOI:** 10.1111/cns.70333

**Published:** 2025-03-19

**Authors:** Yuyang Zhou, Qilin He, Guanglong Huang, Pei Ouyang, Hai Wang, Jiapeng Deng, Pengyu Chen, Xuan Liang, Zhisheng Hong, Xian Zhang, Songtao Qi, Yaomin Li

**Affiliations:** ^1^ Department of Neurosurgery, Institute of Brain Disease, Nanfang Hospital Southern Medical University Guangzhou Guangdong China

**Keywords:** GBM, imaging, microenvironment, PBZ, physics, SMR

## Abstract

**Background:**

Glioblastoma (GBM) is one of the most aggressive primary brain tumors in adults. Over 95% of GBM patients experience recurrence in the peritumoral brain tissue or distant regions, indicating the presence of critical factors in these areas that drive tumor recurrence. Current clinical treatments primarily focus on tumor cells from the tumor core (TC), while the role of neoplastic cells beyond the TC has been largely neglected.

**Methods:**

We conducted a comprehensive review of existing literature and studies on GBM, focusing on the identification and characterization of questionable cells (Q cells). Advanced imaging techniques, such as diffusion tensor imaging (DTI), magnetic resonance spectroscopy (MRS), and positron emission tomography (PET), were utilized to identify Q cells beyond the tumor core. We also analyzed the functional properties, cellular microenvironment, and physical characteristics of Q cells, as well as their implications for surgical resection.

**Results:**

Our review revealed that Q cells exhibit unique functional attributes, including enhanced invasiveness, metabolic adaptations, and resistance mechanisms. These cells reside in a distinct cellular microenvironment and are influenced by physical properties such as solid stress and stiffness. Advanced imaging techniques have improved the identification of Q cells, enabling more precise surgical resection. Targeting Q cells in therapeutic strategies could significantly reduce the risk of GBM recurrence.

**Conclusion:**

The presence of Q cells in the peritumoral brain zone (PBZ) and beyond is a critical factor in GBM recurrence. Current treatments, which primarily target tumor cells in the TC, are insufficient to prevent recurrence due to the neglect of Q cells. Future research should focus on understanding the mechanisms influencing Q cells and developing targeted therapies to improve patient outcomes.

## Graphical abstract

Located beyond the tumor core, questiona cells (Q cells) exhibit high invasiveness, metabolic adaptations, and drug resistance, driving tumor recurrence despite aggressive treatment. Their physical properties, such as low solid stress and soft tissue stiffness, further enhance their migratory and invasive capabilities. Advanced imaging techniques (e.g., DTI, MRS, DWI, PET) enable their detection, while interactions with the tumor microenvironment (e.g., TAMs, Tregs, fibroblasts) present new therapeutic challenges and opportunities.

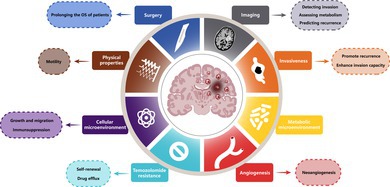

## 1 Introduction

Glioblastoma (GBM) is the most lethal primary brain tumor in adults [1]. Despite aggressive multimodal treatments, including surgery, radiotherapy, and temozolomide (TMZ) chemotherapy, the median survival post‐diagnosis remains 14–16 months [2]. Recent advancements in GBM treatment have extended the median survival to 20.9 months [3]. Following GBM resection, nearly 95% of patients experience recurrence in the peritumoral tissue, suggesting that factors in these regions are closely linked to tumor recurrence [4]. The hypothesis that GBM recurs in the peritumoral brain zone (PBZ), defined as the brain region surrounding the TC that appears hyperintense on T2‐FLAIR MRI but lacks enhancement on T1‐gadolinium‐enhanced MRI, has gained support due to increasing research in this area [5]. GBM cells exhibit high invasiveness by infiltrating peritumoral tissues and diffusing over long distances in the brain, contributing to the failure of glioma treatments [6]. Additionally, low levels of GBM driver mutations and distant recurrence have been detected in normal brain tissue far from the TC, suggesting that GBM cells may exist beyond the PBZ. Therefore, we classify tumor cells outside the contrast‐enhancing (CE) TC as Q cells and propose that these cells are responsible for tumor recurrence.

To identify Q cells beyond the enhancement zone, we can utilize stained biopsy samples and potential biomarkers, including cell number (diffusion MR and MRS), angiogenesis (perfusion MR), metabolism (PET and MRS), and cell proliferation (PET and MRS) [6]. Most novel treatments for recurrent glioblastomas have been developed using TC neoplastic cells as models, often neglecting Q cells [7]. The lack of focus on Q cells may explain the limited efficacy of these therapies in clinical settings. Q cells exhibit heterogeneity and plasticity, characteristics shared with GBM cells. Q cells in the PBZ display diverse phenotypes and can transition among the four subtypes: proneural (PN), mesenchymal (MES), classic (CL), and neural (NL) [8]. The unique functional attributes of Q cells, such as their metabolic microenvironment, angiogenesis, drug resistance, and invasive potential, are strongly linked to GBM recurrence [9, 10, 11, 12]. Q cells reside in a unique cellular microenvironment, distinct from TC neoplastic cells, where interactions with other cells are critical for tumor formation and recurrence [13]. Additionally, the unique physical properties of the region beyond the TC can influence Q cell behavior, and disease progression may be modulated by altering these physical attributes [14, 15, 16]. During surgery, extensive resections are thought to more effectively eliminate Q cells and improve patient survival [17]. This review evaluates Q cells across five key dimensions: imaging, functional properties, cellular microenvironment, physical characteristics, and surgical implications. Additionally, it aims to explore potential therapeutic strategies by deepening our understanding of Q cells.

## 2 Identifying Q Cells in Imaging

MRI is the preferred imaging modality for diagnosing GBM due to its noninvasive nature, high resolution, and superior soft tissue contrast. However, standard MRI sequences have limitations in precisely delineating tumor invasion boundaries, prompting the development of advanced imaging techniques to assess tumor infiltration within the PBZ. Diffusion tensor imaging (DTI) is highly effective in detecting even the most subtle alterations in white matter caused by GBM invasion. Studies have shown a strong correlation between tumor invasion and increased isotropic components in DTI imaging [18]. MRS imaging of the DTI‐defined GBM invasion zone revealed increased levels of Cho/Cr, Cho/NAA, and Glx/Cr, while NAA/Cr levels were reduced [19]. Elevated DWI and ADC signals, reflecting water diffusion, were observed in the PBZ and are thought to indicate peritumoral infiltration of Q cells [20, 21]. Perfusion MRI reliably indicates Q cell infiltration, as these cells often migrate along blood vessels within and beyond the PBZ. A voxel‐to‐voxel comparison between MRI and histology revealed an inverse relationship between Q cell infiltration and perfusion in the PBZ [22]. In PET imaging, higher uptake of 11C‐methionine compared to 18F‐fluorodeoxyglucose in the normal brain tissue suggests tumor infiltration in the PBZ (Table 1) [25]. Overall, advancements in imaging technologies enhance our understanding of Q cells, improving clinical diagnosis and patient outcomes [43].

**TABLE 1:** Summary of Q cell features.

		Q cells feature	Reference
Imaging	DTI	Positive correlation between Q cell invasion and augmentation of isotropic components	[18]
	MRS	Cho/Cr, Cho/NAA, Glx/Cr increase, NAA/Ce decrease	[23]
	DWI/ADC	Elevated signals are associated with peritumoral infiltration of Q cells	[20, 21, 24]
	Perfusion MRI	Q cells migrated along the artery beyond the TC; Q cell infiltration was negatively correlated with perfusion	[22]
	PET	Greater uptake of ^11^C‐methionine beyond the TC indicates the presence of Q cell infiltration	[25]
Functional characteristics	Invasiveness	Q cells have MES subtypes and can be converted from PN to MES subtypes by PMT; Q cells characterized by MES subtype are highly invasive and promote GBM recurrence	[8, 26, 27]
		High expression of miR‐126, miR‐369‐5p, and miR‐487b in Q cells enhances Q cell invasion capacity	[12]
	Metabolic microenvironment	The metabolic microenvironment around Q cells is characterized by elevated levels of fatty acids, with Q cells of the PN subtype exhibiting a higher concentration of very long‐chain fatty acids (VLCFAs) and polyunsaturated fatty acids (PUFA)	[9, 28]
		Q cells have a glycolytic metabolic state but are weaker than TC neoplastic cells	[26]
		The release of glutamate from Q cells leads to the production of ROS and further induces the expression of the systemic xCT transporter leading to the synthesis of GSH	[29, 30, 31]
	Angiogenesis	Q cells have the environment and ability to stimulate blood vessel formation	[10, 32, 33, 34]
	TMZ resistance	MGMT, FADS2 promotes TMZ resistance in Q cells	[11, 35]
Cellular microenvironment	Immune cells	The environment in which Q cells reside is rich in microglia, and crosstalk between Q cells and microglia can contribute to Q cell invasion	[36, 37, 38]
		Q cells possess the potential to form an immunosuppressive microenvironment	[34]
	Glial cells	Co‐localization of oligodendrocyte lineage cells, macrophages, and microglia to enhance stemness gene expression in Q cells	[13]
		Astrocyte‐Q cell GJ communication promotes Q cell invasiveness	[39]
Physical properties	Solid stress	Lower solid stress in the PBZ increases miR‐548 expression and further facilitates the motility of Q cell	[16, 40]
	Stiffness	Lower tissue stiffness outside the TC increases the invasive and migratory behavior of Q cells and further prompts a metabolic change towards glycolysis in Q cells	[41, 42]
Surgery		SMR allows for the removal of more Q cells without compromising the patient's postoperative complications and prolonging the OS of patients	[7]

Identifying Q cells through advanced imaging has significant implications for clinical decision‐making in GBM treatment. DTI enhances the accuracy of tumor‐directed surgery, enabling maximal tumor cytoreduction, and its use in radiation therapy planning allows for customized clinical target volume margins [18]. Increased rCBV sites detected by DSC‐MRI may predict tumor progression and response to anti‐vascular therapies [44]. A distinct hyperintense lesion in a non‐enhancing peritumoral region on DWI may indicate early local or distant recurrence. Additionally, a hyperintense lesion in the PBZ on DWI is a key radiological feature for distinguishing GBM from differential diagnoses, including malignant lymphomas and metastatic brain tumors [20, 21]. As imaging technologies advance, their role in guiding clinical interventions will become increasingly vital, offering new opportunities to enhance patient survival and quality of life.

## 3 Functional Characteristics

### 3.1 Invasiveness

Spatial heterogeneity is an important feature of GBM. The Q cells beyond the TC have the characteristics of the NL and PN subtypes, whereas the tumor cells in the TC have the characteristics of the MES subtype, which is associated with a poorer prognosis and is characterized by greater invasiveness than other subtypes [8, 45]. The proneural‐mesenchymal transition (PMT), a critical mechanism that enhances cancer cell invasiveness and treatment resistance [45], was observed in Q cells. Following ionizing radiation (IR) exposure, the CD133 + PN subtype of Q cells undergoes a C/EBP‐β‐dependent transition to a CD109 + MES subtype, highlighting a mechanism underlying tumor recurrence post‐radiotherapy [8]. Another study demonstrated that Q cells in the peritumoral brain zone (PBZ) are enriched with MES subtypes, suggesting that their infiltration into adjacent brain tissues may significantly contribute to GBM recurrence [26].

Q cells display significant interpatient heterogeneity. Differential expression of invasion‐related markers in the PBZ correlates with disease progression and prognosis. In the PBZ, an elevated periphery/core (P/C) ratio of CD44, a glycoprotein linked to GBM proliferation and invasion, was associated with increased resistance to bevacizumab (Bev) therapy, accelerated tumor progression, and poorer survival outcomes [46]. Additionally, bioinformatics analysis revealed that elevated expression of vascular endothelial growth factor A (VEGFA) and C‐X‐C motif chemokine ligand 8 (CXCL8) in the PBZ is linked to GBM recurrence [47].

Numerous invasion‐related differentially expressed genes (DEGs) were identified between Q cells and TC neoplastic cells. DEGs associated with cell migration (e.g., DHRS9, IPCEF1, and TNR), interstitial matrix invasion (e.g., ATP1A2 and PRODH), anti‐apoptotic processes (e.g., BCL XL and PEA‐15), and stem cell markers (e.g., GD3 ganglioside and NG2 proteoglycan) were up‐regulated in the PBZ. Conversely, DEGs associated with pro‐inflammatory processes (RAGE, P2X7R, COX2, NOS2, and PTX3) were found to be down‐regulated in the PBZ [32, 33, 36, 48, 49, 50, 51]. On the other hand, genes that regulate cellular proliferation, motility, and growth (CSRP2, TAZ, ID3, DTNA, HIST2H2AA, EGFR, IGFBP5, VCAM1, and CD99) were upregulated in the PBZ in comparison to the normal brain tissue [52].

MicroRNAs (miRNAs) and long noncoding RNAs (lncRNAs), key regulators of GBM progression [53, 54], exhibit differential expression between the TC and PBZ. Overexpression of 23/768 miRNAs was detected in Q cells and 22/768 miRNAs in TC neoplastic cells. Silencing the top three overexpressed miRNAs (miR‐126, miR‐369‐5p, and miR‐487b) in Q cells effectively inhibited GBM cell invasion, underscoring their functional role in GBM invasion and demonstrating the greater invasive potential of Q cells compared to TC neoplastic cells [12]. A highly expressed lncRNA IncHERG in GBM was found, and the IncHERG could serve as a sponge for tumor suppressor miR‐940. The up‐regulation of miR‐940 in the PBZ, relative to TC tissues, correlates with changes in IncHERG levels, further highlighting the critical role of lncHERG as a competing endogenous RNA for miR‐940 in GBM [55].

**FIGURE 1:**
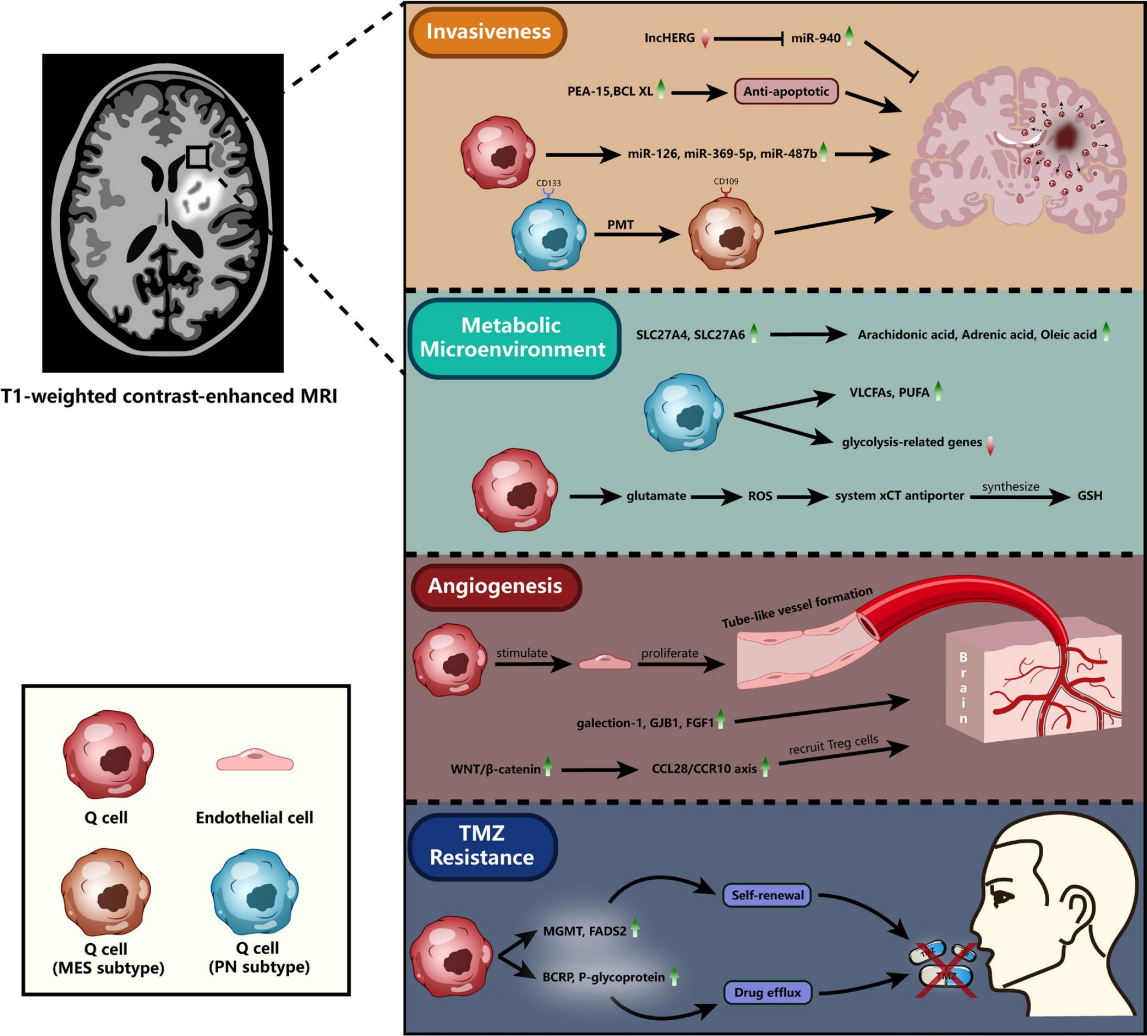
Functional characterization of Q cells beyond the tumor core. Q cells that are situated beyond the CE zone possess particular functional attributes. PMT is capable of converting Q cells into more invasive subtypes of MES; furthermore, their invasive capability is enhanced by the overexpression of miR‐940, miR‐126, miR‐369‐5p, miR‐487b, PEA‐15, and BCL XL. In comparison to the TC microenvironment, the Q cell microenvironment is richer in a variety of fatty acids (including arachidonic acid, Adrenic acid, oleic acid, VLCFA, and PUFA). Glutamate release by Q cells can further stimulate GSH synthesis. Down‐regulation of glycolysis‐related genes in the PN subtype of Q cells results in weaker glycolysis than in TC tumor cells. Galectin‐1, GJB1, FGF1, and the CCL28/CCR10 axis, which are all upregulated, promote angiogenesis in the region containing Q cells. In the interim, Q cells facilitate the formation of tubular blood vessels through the promotion of proliferation of endothelial cells. By increasing Q cell self‐renewal capacity, high expression of MGMT and FADS2 can confer TMZ resistance; conversely, high expression of BCRP and P‐glycoprotein promotes drug efflux and TMZ resistance.

In summary, substantial evidence indicates that the expression of invasion‐related substances and pathways is elevated in the PBZ (Figure 1 and Table 1). This highlights the presence of Q cells in the peritumoral region and underscores the PBZ as a critical site for tumor malignant progression and recurrence.

### 3.2 Metabolic Microenvironment

Due to the differences in gene expression and surrounding microenvironments, the TC and PBZ of GBM have different metabolic characteristics [9]. In the following sections, we will elucidate the metabolic profiles of regions outside the TC based on metabolite types and highlight their potential as therapeutic targets against Q cells.

#### 3.2.1 Fatty Acid Metabolism

The expression levels of various fatty acids (FAs), including ω‐6 fatty acids such as arachidonic acid, adrenic acid, and oleic acid, were significantly higher in the PBZ compared to the TC [9]. As members of the solute carrier family 27 (SLC27), very‐long‐chain acyl‐CoA synthetases SLC27A4 and SLC27A6, which are associated with the transport of FAs, were expressed higher in the PBZ compared to the TC [56]. Furthermore, there are differences in FA metabolism among different subtypes of GBM. The overall level of triacylglycerols increased and glycerophospholipids decreased in the MES subtypes of GBM, while very‐long‐chain fatty acids (VLCFAs) and glycerophospholipids with polyunsaturated fatty acid (PUFA) side chains were enriched in the PN subtypes [28]. These differences suggest distinct FA metabolic profiles between Q cells with PN characteristics in the PBZ and MES subtype neoplastic cells in the TC (Figure 1 and Table 1).

#### 3.2.2 Glucose Metabolism

Glycolysis, a glucose metabolic pathway critical for energy production, is less active in the PBZ compared to the TC. Endothelial cells (ECs) in the TC exhibited upregulation of glycolysis, citric acid cycle, and oxidative phosphorylation gene expression, reflecting the high energy demands for angiogenesis in the TC microenvironment [57]. Glycolysis‐related gene expression was significantly downregulated in the PBZ compared to the TC, although Q cells within the PBZ maintained a glycolytic metabolic profile (Figure 1 and Table 1) [26]. Since GBM recurrence is thought to result from the transformation of Q cells into TC neoplastic cells [26], targeted therapies inhibiting the high glycolytic activity of TC neoplastic cells may prevent this transition and reduce recurrence risk.

#### 3.2.3 Amino Acid Metabolism

Unlike the TC, the PBZ shows reduced expression of glutamine (Gln) and its metabolite glutathione (GSH) [9], both of which contribute to chemoradiotherapy resistance and oxidative stress tolerance [58]. GBM releases glutamate, a product of glutamine catalyzed by glutaminase (GLS), into the extracellular space. Extracellular glutamate levels in the PBZ are higher than that in the normal brain tissue [59]. Elevated extracellular glutamate levels can induce reactive oxygen species (ROS) production and upregulate the system xCT antiporter, a cysteine/glutamate transporter component, to promote GSH synthesis (Figure 1 and Table 1) [29, 58]. In GBM patients with refractory seizures, significantly elevated extracellular glutamate levels in the PBZ were observed. This suggests that PBZ glutamate measurement via magnetic resonance spectroscopy (MRS) may predict refractory epilepsy and guide antiepileptic drug use [60]. Therapies targeting glutaminase (GLS) to regulate glutamate metabolism are effective in GBM [61], highlighting their potential for PBZ‐targeted treatment.

#### 3.2.4 Nucleotide Metabolism

Purine and pyrimidine levels, essential for nucleotide and deoxyribonucleotide synthesis, are lower in the PBZ than in the TC [9]. Elevated purine and pyrimidine metabolite levels in the TC are associated with its high energy demands and hypoxic microenvironment [62]. Purine metabolites, particularly guanylates, contribute to radiation resistance in GBM by promoting GTP synthesis [63]. Inhibition of the de novo pyrimidine synthesis pathway, one of the two primary purine and pyrimidine metabolic pathways, has been shown to suppress glioblastoma stem cell (GSC) growth [64]. These findings underscore the distinct purine and pyrimidine metabolic profiles in the PBZ and highlight the potential of nucleotide metabolism‐targeted therapies to prevent Q cell transformation into TC neoplastic cells.

### 3.3 Angiogenesis

Angiogenesis is widely recognized as a critical driver of glioblastoma (GBM) progression. The expression of angiogenesis markers (HIF1α, HIF2α, VEGF, VEGFR1, and VEGFR2) was detected in both TC and PBZ tissue when the expression of several angiogenesis‐related factors was assessed in GSCs isolated from TC and PBZ tissue. Furthermore, in vitro tube formation assays further revealed that Q cells isolated from the PBZ promote endothelial cell (EC) proliferation and tube‐like vessel formation [10]. Both the CCL28/CCR10 axis and its proximal signaling pathway WNT/β‐catenin are upregulated in the PBZ, which promotes tumor angiogenesis by recruiting regulatory T cells (Treg cells) [34, 65, 66]. Additionally, substances associated with angiogenesis, including galectin‐1, GJB1, and FGF1, are significantly overexpressed in the PBZ [32, 33]. This finding provides evidence for the presence of neoangiogenesis in the PBZ.

However, angiogenesis is generally more pronounced in the TC than in the PBZ. Reduced expression of angiogenesis‐related genes (e.g., VEGF, ANGPT2, HIF, PDGF, and ICAM1) in the PBZ compared to the TC suggests less robust angiogenesis in the PBZ [52, 67]. In conclusion, both regions within and beyond the TC contain factors that promote angiogenesis, and angiogenesis is typically more pronounced in the TC than in the PBZ (Figure 1 and Table 1).

## 4 Temozolomide Resistance

Standard TMZ treatment is considered a significant factor contributing to GBM recurrence in the PBZ following resection [68]. The mechanisms driving TMZ resistance in Q cells are increasingly understood (Figure 1). Molecules associated with cell self‐renewal and DNA repair were found in the PBZ. Expression levels of O6‐methylguanine methyltransferase (MGMT), stearoyl‐coenzyme A desaturase (SCD), and fatty acid desaturase 2 (FADS2) were higher in the PBZ than in the TC [11, 35]. FADS2 is critical for Q cell viability and self‐renewal, while elevated SCD expression correlates with increased TMZ resistance (Table 1) [35, 69].

At the same time, the blood–brain barrier (BBB) and its main component play a key role in regulating drug permeability and drug resistance in GBM [70]. GBM activates STAT3 in BBB cells via IL‐6 release, downregulating ATP‐binding cassette (ABC) transporters and tight junction (TJ) proteins. This increases BBB permeability and facilitates tumor infiltration [71]. However, BBB disruption in gliomas primarily occurs in the TC due to elevated VEGF expression and angiogenesis in hypoxic regions rather than in the PBZ. This may explain why tumors infiltrate from the central GBM region to the periphery through permeable vessels, while the intact BBB in the PBZ shields tumor cells from drug effects [72]. Several key blood–brain‐barrier (BBB) transporters, including ABCB1 and breast cancer resistance protein 1 (BCRP1), also known as ABCG2, were highly expressed in the ECs located in the PBZ [11, 33, 57]. P‐glycoprotein encoded by ABCB1 and BCRP encoded by ABCG2 together mediated the efflux of xenobiotics, including temozolomide and other low–molecular weight anticancer drugs from the endothelium away from the neuroparenchymal space, which reveals the functional integrity of the blood–brain barrier and the chemotherapy resistance of the PBZ [73].

PDZ‐binding kinase (PBK), a novel PBZ biomarker, is implicated in GBM chemoresistance and is significantly upregulated in the PBZ compared to the TC [74]. PBK inhibition enhances GBM radiotherapy efficacy by downregulating CCNB2, a key cell cycle regulator [75]. However, the role of PBK in TMZ resistance remains unclear.

As shown in Figure 1, Q cells beyond the contrast‐enhancing (CE) zone exhibit unique functional characteristics, including enhanced invasiveness via the proneural‐mesenchymal transition (PMT), metabolic adaptations (e.g., increased fatty acid metabolism and glutamate release), and upregulated angiogenesis markers (e.g., galectin‐1, GJB1, and FGF1).

## 5 Cellular Microenvironment

**FIGURE 2:**
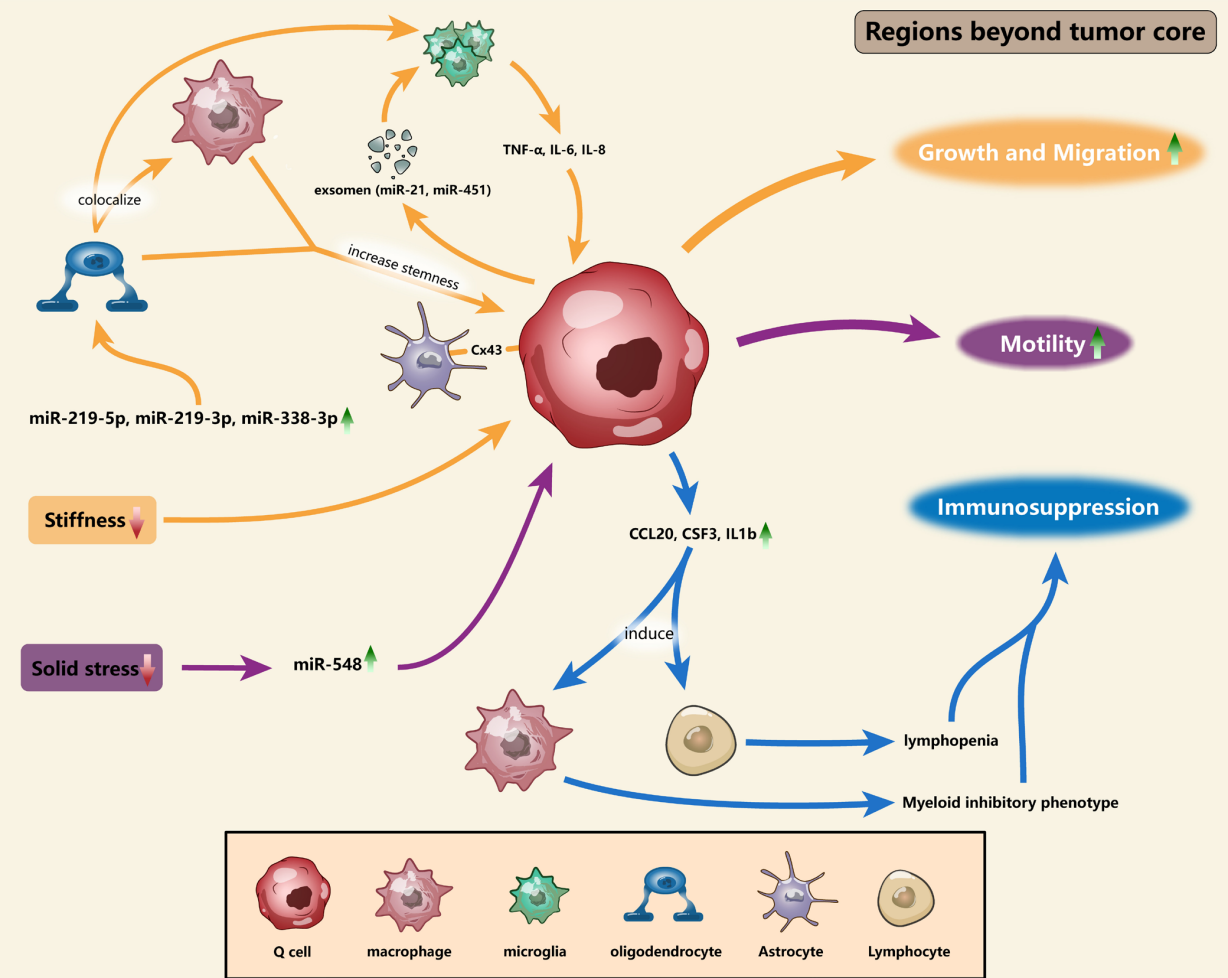
Influence of cellular microenvironment and physical properties on Q cells. The co‐localization of oligodendrocyte lineage cells, macrophages, and microglial cells in the PBZ is induced by the high expression of three microRNAs (miR‐219‐5p, miR‐219‐3p, and miR‐338‐3p); meanwhile, this co‐localization subsequently stimulates the expression of stemness genes in Q cells. Q cells' proliferation and migration are concurrently stimulated by interactions with microglia and astrocytes. High expression of CCL20, CSF3, and IL1b by Q cells induces lymphopenia as well as differentiation of the monocyte phenotype into a myelosuppressive phenotype, further contributing to the formation of an immunosuppressive microenvironment around Q cells. The mobility and migration of Q cells are facilitated by environmental attributes such as low solid stress and stiffness.

Q cells are surrounded by a distinct cellular microenvironment compared to TC neoplastic cells (Figure 2 and Table 1). Tumor‐associated macrophages (TAMs), the most abundant immune cells in the GBM microenvironment, comprise microglia and bone marrow‐derived myeloid cells [76]. Analysis of TAMs in the PBZ and TC revealed that TC cells predominantly expressed macrophage‐associated genes, while PBZ cells expressed microglia‐associated genes, indicating that tumor‐infiltrating macrophages and resident microglia preferentially localize to the TC and PBZ, respectively [36]. TAMs secrete transforming growth factor‐beta (TGF‐β), which suppresses cytotoxic T cell activity, promotes lymphocyte depletion, and enhances M2 macrophage polarization. M2 macrophages secrete chemokines (CCL2, CCL5, CCL20, and CCL22), which recruit regulatory T cells and inhibit CD4+ and CD8+ effector cells, natural killer (NK) cells, and dendritic cells (DCs). Additionally, TAMs release matrix metalloproteinases (MMP2 and MMP9), which degrade the extracellular matrix (ECM) and facilitate tumor cell invasion and migration [77]. The potential crosstalk between APOC1 + CCL3+ microglial cells and neural stem cells in the PBZ tissue plays certain roles in the recurrence of GBM [78]. Microglia enhance tumor cell invasion through the secretion of TGF‐β and regulation of MMP2, which degrades the extracellular matrix (ECM). GBM cells release exosomes containing miR‐21 and miR‐214‐5p, which stimulate microglial proliferation and the secretion of inflammatory factors (TNF‐α, IL‐6, and IL‐8), thereby enhancing GBM cell growth and migration [37, 38]. These findings suggest that the interaction between microglia and Q cells plays a role in GBM recurrence. Additionally, immune cells outside the TC create an immunosuppressive microenvironment. PD‐1 + CD8+ T cells, Foxp3+ regulatory T cells, and CD163+ macrophages were more abundant in the TC compared to the PBZ [79]. IQ cells isolated from the PBZ exhibit upregulation of genes (e.g., CCL20, CSF3, and IL1b) that induce lymphopenia and promote monocyte differentiation into an immunosuppressive myeloid phenotype, suggesting an immunosuppressive role in the PBZ [34].

Glial cells beyond the TC have been found to play an important role in the recurrence and progression of GBM. Three miRNAs (miR‐219‐5p, miR‐219‐3p, and miR‐338‐3p) are highly expressed in the PBZ and are associated with oligodendrocyte differentiation. This leads to the colocalization of oligodendrocyte lineage cells, macrophages, and microglia in the PBZ, enhancing stemness gene expression in Q cells [13]. Connexin 43 (Cx43), as a principal astrocytic gap junction (GJ) protein, was found localized to the PBZ, revealing that Q cells‐astrocyte GJ communication in the PBZ is a driving force for invasion [39]. VAV1, a GDP/GTP exchange factor for Rho/Rac proteins, is expressed in astrocyte‐like cells located peritumorally or perivascularly outside the tumor. Moreover, VAV1 expression may contribute to the neoplastic process in GBM and potentially induce a synergistic response in GBM cells [80]. A novel platelet‐derived growth factor receptor alpha (PDGFRα) + subset of glial fibrillary acidic protein (GFAP) + astrocytes was found in the PBZ, and their presence is associated with shorter median survival in GBM patients [81].

Mesenchymal stem cells (MSCs) are also present in the tumor periphery. Glioma‐associated mesenchymal stem cells (GA‐MSCs) promote glioma progression by enhancing proliferation, invasion, and angiogenesis. They release IL‐6 and exosomes (including miR‐1587) to sustain the stemness and proliferation of glioma stem cells (GSCs). GA‐MSCs promote invasion via the C5a/p38 MAPK/ZEB1 pathway and CCL2/JAK1‐mediated extracellular matrix remodeling. Additionally, GA‐MSCs induce angiogenesis by secreting factors like SDF‐1/CXCL12 and HGF [82]. Glioblastoma‐derived cancer‐associated fibroblasts (CAFs) promote the migration and invasion of malignant cells, while CAF‐derived fibronectin 1 (FN1) further enhances these properties. CAF‐derived extra structural domain A (EDA) fibronectin variations are linked to the production of M2 macrophage polarization via interaction with TRL4 in the GBM [83].

## 6 Physical Properties

### 6.1 Solid Stress

Solid stress, defined as the compressive and tensile mechanical forces exerted by solid components in tissues, significantly influences tumor growth, invasion, metastasis, and treatment efficacy [84]. Solid stress in GBM gradually decreases from a peak value of 210 Pa at the tumor margin to the PBZ [40]. It was observed that the migratory capability of glioblastoma multiforme (GBM) cells reached its maximum at 23 Pa under solid stress conditions spanning from 0 to 115 Pa. Additionally, low levels of solid stress upregulate miR‐548, which may enhance GBM cell motility [16]. In the PBZ, both the radial (compressive) and circumferential (tensile) components of solid stress exhibit a gradient decrease. In vivo, solid stress induces neuronal dysfunction and vascular perfusion injury, while systemic lithium therapy protects neurons from mortality and neurological dysfunction caused by solid stress. These studies demonstrate that solid stress in the PBZ enhances Q cell invasiveness and therapies targeting solid stress hold significant therapeutic potential (Figure 2 and Table 1).

### 6.2 Fluid Pressure

Interstitial fluid pressure (IFP) refers to the biophysical manifestation of the pressure gradient, typically between a capillary and lymphatic drainage. In GBM, elevated vascular permeability, driven by tumor‐mediated angiogenesis and dysplastic vessels, leads to increased IFP [85]. Studies on mammary adenocarcinoma show that IFP is nearly uniform throughout the tumor but sharply decreases at the tumor edge [86]. At the tumor edge, IFP drops to the normal range of −3 to 3 mmHg, resulting in fluid leakage into the peritumoral region at rates of 2–50 μm/s. In addition, abnormal fluid flow is prevalent in brain cancers, and the flow into healthy tissues will lead to peritumoral brain edema, intracranial pressure, and invasion of cancerous cells into the PBZ [87]. IFP upregulates plasminogen activator (uPA), matrix metalloproteinases (MMPs), and epithelial‐mesenchymal transition (EMT) markers in GBM cells via mechanosensing by caveola‐forming proteins (caveolin‐1, CAVIN1), enhancing invasiveness [88]. Collectively, IFP‐driven fluid flow enhances Q cell invasiveness and is a key driver of Q cell infiltration beyond the TC.

### 6.3 Stiffness

Stiffness, also known as elasticity or rigidity, has been proven to promote tumor initiation, progression, and invasion [89]. Kren et al. [14] measured the mechanical characteristics of GBM tumor (more than 60% tumor cells) and non‐tumor (no tumor cells detected) tissues by indentation experiments and found that there was no statistically significant difference in elasticity values between the two types of tissues and that the non‐tumor tissue showed a slightly more rapid stress relaxation behavior than the tumor tissue. On the other hand, A. Sohrabi et al. [41] demonstrated that the PBZ has lower stiffness than the TC, and this softer environment induces a glycolytic shift in Q cells, enhancing their migratory activity. The soft environmental stiffness can enhance the invasive capacity of tumor cells, whereas the stiffer microenvironment can prompt the adoption of the MES cell shape, leading to increased expression of intracellular cytosolic ROS and decreased expression of mitochondrial ROS [90]. Recurrent GBM cultured in a specific substrate stiffness (500 Pa) was found to have higher expression of the ECM proteins (e.g., collagen, MMP2, and MMP9) as well as greater tumorigenicity and recurrent disease progression in vivo [91]. These studies suggest that PBZ stiffness promotes Q cell invasion and drives GBM recurrence (Figure 2 and Table 1).

As shown in Figure 2, the co‐localization of oligodendrocyte lineage cells, macrophages, and microglial cells in the PBZ is driven by the high expression of miR‐219‐5p, miR‐219‐3p, and miR‐338‐3p. This interaction upregulates stemness genes in Q cells, enhancing their proliferation and migratory capacity. Additionally, low solid stress and stiffness in the PBZ microenvironment further facilitate Q cell motility and invasion.

## 7 Surgery

In GBM surgical resection, the extent of resection (EOR) is strongly associated with postoperative patient survival [92]. The primary goal of GBM surgery is complete tumor removal, as visualized on T1‐contrast‐enhanced MRI [92]. Even after complete removal of the tumor region on imaging, residual Q cells outside the TC often necessitate postoperative adjuvant therapy [2]. Supramaximal resection (SMR), which involves the complete removal of abnormal FLAIR signals, significantly improves overall survival (OS) in glioma patients [93, 94]. Meta‐analyses and systematic reviews demonstrated that patients with SMR have longer progression‐free survival (PFS) and OS than patients with gross total resection (GTR), with no significant difference in the postoperative complication rate (Table 1) [17, 95].

While SMR definitively prolongs OS, the optimal resection extent remains debated. SMR between 20% and 60% significantly improves OS, but benefits plateau beyond 60% [96]. The optimal SMR threshold for maximum OS varies with the degree of GBM invasion. The SMR with significant OS benefit in nodular tumors and highly diffuse tumors was pointed out, which were defined by the tumor proliferation rate (ρ)/diffusion rate (D) ratio, which is 10% to 20% and 30% to 90%, respectively [97]. Recent advances enable voxel‐level differentiation between Q cells and vasogenic edema in the PBZ, refining the effective scope of SMR [98].

Neurosurgeons prioritize maximizing resection extent while ensuring patient safety, driving the development of advanced methodologies to optimize resection. 5‐Aminolevulinic acid (5‐ALA), a fluorescent dye for detecting non‐enhancing tumor lesions, improves PFS and OS in GBM patients by enhancing resection accuracy [99]. Our study demonstrated that the en‐bloc technique, which inhibits tumor invasion, facilitates SMR and prolongs survival in primary GBM [100]. Combining contrast‐enhanced ultrasound (ETUS) with 5‐ALA improves EOR compared to conventional microsurgery [101]. The dual intraoperative visualization approach (DiVA), integrating intraoperative MRI (iMRI) and 5‐ALA, enhances SMR and prolongs survival in GBM patients [102]. Plasmonic‐based nanostructured biosensors, a label‐free system, distinguish tumors from the surrounding tissue via refractive index differences during surgery [103]. With a further understanding of the optimal resection range and the improvement of the technology to identify the boundary of GBM, we emphasize the necessity of SMR in the treatment of GBM and affirm its positive benefits for patients.

As summarized in Table 1, the PBZ is a critical site for GBM recurrence due to the unique characteristics of Q cells. These cells exhibit enhanced invasiveness, metabolic adaptations, and resistance mechanisms, all of which contribute to treatment failure and tumor recurrence. Targeting these features in Q cells may provide new therapeutic opportunities to improve patient outcomes.

## 8 Conclusions

The PBZ is a crucial position for GBM recurrence. Q cells present in the PBZ and beyond subsequent to surgical resection will contribute to treatment resistance and tumor recurrence through a variety of pathways. By leveraging developments in detection technology, it is now possible to identify Q cells beyond the TC with greater precision, thereby facilitating SMR. Unfortunately, there is a lack of comprehensive research on the mechanisms influencing Q cells. Most current radiotherapy and chemotherapy interventions rely on the study of TC neoplastic cells. However, the limited understanding of Q cells has resulted in the suboptimal performance of conventional treatments. In general, we hold the belief that future research should concentrate on the areas where Q cells are abundant and that the key to developing effective treatments for GBM will be to target Q cells.

## Author Contributions

Y.Z., Y.L., S.Q., X.Z., and Q.H. conceived and designed this review; Y.Z., Q.H., Z.H., and G.H. drafted the manuscript; P.O., H.W., X.L., J.D., and P.C. collected the data; Y.Z., S.Q., and Y.L. revised the manuscript. All authors read and approved; the present version of the manuscript to be published. The authors take full responsibility for this article.

## Ethics Statement

The authors have nothing to report.

## Consent

The authors have nothing to report.

## Conflicts of Interest

The authors declare no conflicts of interest.

## Data Availability

Data sharing was not applicable to this article as no datasets were generated or analysed during the current study.
